# *In vitro* susceptibility of *Trypanosoma cruzi* discrete typing units (DTUs) to benznidazole: A systematic review and meta-analysis

**DOI:** 10.1371/journal.pntd.0009269

**Published:** 2021-03-22

**Authors:** Andrea Vela, Marco Coral-Almeida, Denis Sereno, Jaime A. Costales, Christian Barnabé, Simone Frédérique Brenière

**Affiliations:** 1 Institut de recherche pour le développement (IRD), UMR INTERTRYP IRD-CIRAD, University of Montpellier, Montpellier, France; 2 Centro de Investigación para la Salud en América Latina (CISeAL), Escuela de Ciencias Biológicas, Universidad Católica del Ecuador, Quito, Ecuador; 3 One Health Research group, Facultad de Ciencias de la salud, Universidad de las Américas-Quito, Calle de los Colimes y Avenida De los Granados, Quito, Ecuador; Universidad de Buenos Aires, ARGENTINA

## Abstract

**Background:**

Chagas disease, a neglected tropical disease endemic to Latin America caused by the parasite *Trypanosoma cruzi*, currently affects 6–7 million people and is responsible for 12,500 deaths each year. No vaccine exists at present and the only two drugs currently approved for the treatment (benznidazole and nifurtimox), possess serious limitations, including long treatment regimes, undesirable side effects, and frequent clinical failures. A link between parasite genetic variability and drug sensibility/efficacy has been suggested, but remains unclear. Therefore, we investigated associations between *T*. *cruzi* genetic variability and *in vitro* benznidazole susceptibility via a systematic article review and meta-analysis.

**Methodology/Principal findings:**

*In vitro* normalized benznidazole susceptibility indices (LC_50_ and IC_50_) for epimastigote, trypomastigote and amastigote stages of different *T*. *cruzi* strains were recorded from articles in the scientific literature. A total of 60 articles, which include 189 assays, met the selection criteria for the meta-analysis. Mean values for each discrete typing unit (DTU) were estimated using the meta and metaphor packages through R software, and presented in a rainforest plot. Subsequently, a meta-regression analysis was performed to determine differences between estimated mean values by DTU/parasite stage/drug incubation times. For each parasite stage, some DTU mean values were significantly different, e.g. at 24h of drug incubation, a lower sensitivity to benznidazole of TcI vs. TcII trypomastigotes was noteworthy. Nevertheless, funnel plots detected high heterogeneity of the data within each DTU and even for a single strain.

**Conclusions/Significance:**

Several limitations of the study prevent assigning DTUs to different *in vitro* benznidazole sensitivity groups; however, ignoring the parasite’s genetic variability during drug development and evaluation would not be advisable. Our findings highlight the need for establishment of uniform experimental conditions as well as a screening of different DTUs during the optimization of new drug candidates for Chagas disease treatment.

## Author summary

Although a century has elapsed since Chagas disease discovery, only two drugs, benznidazole and nifurtimox, are approved for its treatment. These drugs have significant safety and efficacy limitations and their specific modes of action are still poorly understood. Moreover, the causative agent, the protozoan parasite *Trypanosoma cruzi*, displays high genetic variability, which is suspected to affect clinical manifestations and disease outcome. With these facts in mind, we aimed to elucidate the role of the parasite’s genetic background in benznidazole treatment efficacy. Our results unveiled interesting differences in the sensitivity to benznidazole between some *T*. *cruzi* genotypes, which suggests genetic variability could influence patient cure rates. Additionally, they show the need for uniformity in experimental conditions during laboratory analysis of new drug candidates for Chagas disease treatment, and they highlight the importance of screening diverse *T*. *cruzi* strains from different genetic background as part of the process of drug evaluation and optimization.

## Introduction

Chagas disease (CD) or American trypanosomiasis, a neglected tropical disease, affects 6–7 million people worldwide [1]. CD is lifelong and often lethal, leading to an estimated 12,500 deaths each year [2]. CD’s global economic burden is estimated to be 7.19 billion dollars annually, most of which arises from lost productivity from cardiovascular disease-induced early death [3]. The clinical manifestations are diverse, and progress in two distinct clinical phases. During the acute phase, which comprises the first ~8 weeks after the initial infection, trypomastigotes are present in the blood of infected individuals. However, the disease usually goes unrecognized, because symptoms may be absent or mild [4]. However, ~5% of those infected, especially children, may die during the acute phase [5]. Around 8 weeks post-infection, the immune response clears most parasites, and the chronic phase ensues. Patients seroconvert; however, the vast majority of those infected (~60–70%) remain asymptomatic and without visceral involvement, i.e. in the indeterminate form of the disease [6]. On the other hand, as many as ~30–40% infected individuals will present cardiac or gastrointestinal abnormalities, which may take decades to develop [7].

The causative agent is *Trypanosoma cruzi*, a protozoan parasite with a digenetic life cycle involving hematophagous invertebrate hosts (triatomine bugs) and mammalian hosts. The major transmission route is vectorial, where triatomines defecate on the skin of the vertebrate host during a blood meal or immediately after. The infective metacyclic trypomastigotes present in the feces penetrate the host´s skin through the bite wound, other skin lesions or mucous membranes, and infect a variety of vertebrate host cells (mainly reticuloendothelial, muscular and nervous cells) [8]. Once within the host cells, parasites transform into amastigotes, which multiply by binary fission. After several rounds of replication, the parasites differentiate into trypomastigotes, which in turn rupture the host cell’s plasma membrane and enter the bloodstream, from where they may invade new cells, new organs or may also be taken up by a new triatomine bug. When the latter occurs, the trypomastigotes present in the ingested blood differentiate into epimastigotes, which colonize the triatomine digestive tract, divide by binary fission and migrate to the rectal ampoule, where they become infective metacyclic trypomastigotes. Other less frequent modes of transmission include congenital transmission, blood transfusion, organ transplant, oral contamination, and laboratory accident [9]. The three different forms of the parasite life cycle can be cultured in the laboratory. Epimastigotes are cultured in axenic medium, while trypomastigote and amastigote culture requires infecting mammalian cells *in vitro*.

Nifurtimox and benznidazole, the only drugs available for CD treatment, have been available since the 1960s [1,10,11]. However, their pharmacokinetics and pharmacodynamics are still not completely understood [8]. Nifurtimox is a nitrofuran capable of producing highly toxic reactive oxygen species and free radicals, increasing oxidative stress [12]. It was the first drug used for treatment of CD and its efficacy is greater when provided during the acute phase or when given to children under 14 years of age, where cure rates ranged from 88–100% [13–16]. For adult patients treated during the chronic phase, only 7–8% cure rate was obtained. It remains an alternative drug for patients with benznidazole intolerance [17]. Benznidazole is a nitroimidazole derivative, which has been reported to increase reductive stress and to covalently modify the parasite´s proteins and DNA. It is better tolerated than nifurtimox, has similar chemotherapeutic efficacy and is currently the drug of choice for CD treatment [16]. Treatment is most efficient in the acute phase (80–100% cure rates) and therefore recommended in early stage patients, children up to 18 years of age, women in childbearing age, congenitally acquired cases and in cases of reactivation due to immunosuppression [18]. For patients presenting the indeterminate form of the disease, it is recommended that treatment is offered; however, the benefits of therapy should be weighed against possible toxic side effects [18]. As the CD chronic phase progresses, treatment efficacy has been shown to decrease, (cure rates of a maximum of 60% have been reported) [19]. Treatment of chronic patients suffering cardiomyopathy or gastrointestinal disorders remains controversial: some authors discourage treatment and promote mainly supportive therapy [19], while others consider that treatment attenuates CD progression and prevents electrocardiographic abnormalities and heart failures [20–22]. Both drugs can cause severe adverse effects that can lead to treatment interruption and patient non-compliance; these include nausea, vomiting, weight loss, insomnia, severe dermatitis, peripheral neuropathies and lymphadenopathies, occurring in up to 50% of treated adults [11]. Both drugs are a part of the WHO List of Essential Medicines [23], and in the U.S, benznidazole is the first drug approved by the Food and Drug Administration (FDA) for treatment of CD in children under age 12 [24]. Benznidazole was initially produced and commercialized by Roche, until 2003, when rights were transferred to the Brazilian Pharmaceutical Laboratory of Pernambuco State (LAFEPE), under the supervision of the Brazilian Ministry of Health [25]; In 2012, the Argentinian private laboratories ELEA and MAPRIMED overtook benznidazole production also assuring its availability; Bayer has since renewed production of nifurtimox [25]. Given difficulties in accessing these drugs in many countries, in 2015, it was estimated that at least 80% of CD patients lacked timely diagnosis and treatment [26].

Genetic flow between *T*. *cruzi* strains has been traditionally considered to be absent or scarce; therefore, this species has been deemed primarily clonal [27]. However, recent evidence suggests that meiotic reproduction may be more common than previously appreciated [28,29]. Seven distinct genetic lineages or discrete typing units (DTUs), formally named TcI to TcVI and TcBat are currently recognized within *T*. *cruzi* species based on genotyping [14,30,31]. They differ widely in their geographical distribution and ecological niche [32]. Briefly, TcI, a DTU with high genetic diversity has the widest geographical distribution and predominates in both sylvatic and domestic cycles; TcII of low genetic diversity, is extremely rare in North and Central America, and is mostly related to domestic cycles in the regions south of the Amazon; TcIII and TcIV are also rarely sampled and mostly associated with sylvatic transmission cycles even if they can infect humans; TcV and TcVI, the most recent DTUs resulting from hybridization between strains of DTUs TcII and TcIII or TcIV, are clearly associated with domestic transmission cycles. In Peru, Bolivia and northern Chile, TcV is the most common DTU, while in Argentina and Paraguay both TcV and TcVI can be found [32]. Tcbat, composed of strains initially isolated from bats, was more recently identified as an independent *T*. *cruzi* DTU more related to TcI than to other DTUs [33]; nevertheless, a first human infection with Tcbat was recorded in a 5-year-old female living in a forest area in northwestern Colombia [34]. *T*. *cruzi* strains are heterogeneous regarding most biological characteristics for both the experimental *in vitro* and *in vivo* models, i.e. each strain displays distinct properties in terms of infectivity, metabolic activity, enzymatic expression and, varying levels of *in vitro* drug susceptibilities and natural resistance [35,36]. It has been known for a long time that high variability exists among *T*. *cruzi* strains in terms of susceptibility to benznidazole and nifurtimox [37]. Furthermore, it has been previously suggested that the genetic diversity of the parasite may influence infection evolution, clinical presentation and treatment outcome during CD [38,39]. Nevertheless, a recent systematic review failed to find statistically significant associations between *T*. *cruzi* genotype and chronic clinical outcome, risk of congenital transmission, reactivation and orally transmitted outbreaks [40]. Two other *in vitro* studies using TcI, TcII and TcV DTUs failed to show correlation between benznidazole susceptibility and genetic distances between DTU strains [41,42].

In this context, in order to better evaluate a possible association between benznidazole susceptibility and specific *T*. *cruzi* genetic lineages, we conducted a systematic review and meta–analysis of the *in vitro* benznidazole susceptibility assays available in the literature. The susceptibility to benznidazole among different *T*. *cruzi* DTUs was estimated as half-maximal inhibitory concentration (IC_50_) for epimastigotes and amastigotes, and half-maximal lethal concentration (LC_50_) for trypomastigotes.

## Methods

### Literature search and data collection

A systematic literature search on *in vitro* benznidazole susceptibility of *T*. *cruzi* strains was carried out focusing on the drug response of the different genotypes (DTUs) according to the Prisma statement [43]. Publications were searched in PubMed with no date or language restrictions using the Boolean operator “AND” plus the keywords: “benznidazole AND *cruzi* AND strain (s)”, “benznidazole AND DTU”. After manually removing duplicated publications based on the titles, the remaining abstracts were individually screened. Publications were excluded based on the following criteria: *in vivo* studies, no LC_50_ or IC_50_ values reported for benznidazole, review publications and *in vitro* studies with drugs not including benznidazole. Subsequently, publications were selected on the basis of full-text analysis, according to the availability of the following additional information: standard deviation for benznidazole LC_50_ or IC_50_ values, strain name, DTU of strains studied and, incubation time of the parasite with the drug (time point). Assays available in each selected publication were recorded in a database (Excel file) where the following variables were registered: name of the strain, code of the parasite laboratory clone when it exists, country origin of the strain, DTU to which the strain belongs, parasitic form on which the assay was carried out (epimastigote, trypomastigote or amastigote), drug incubation time, LC_50_ (trypomastigotes) or IC_50_ (amastigotes and epimastigotes) values expressed in μM, standard deviation of LC_50_ or IC_50_ values expressed in μM, number of replicates for each assay, parasite viability determination method, publication year, authors and title of the publication.

### Descriptive analysis of the data

Contingency tables for DTUs and the other qualitative variables were generated by cross tabulation in Microsoft Excel. Figures were calculated for each parasite form and overall assays.

### Statistical analysis

Compiled data (S1 Table) was analyzed via random effects meta-analysis (a formal quantitative statistical analysis of similar experiments or studies) to test for statistically significant differences among DTUs in terms of LC_50_ or IC_50_ mean values. Indeed, the meta-analysis approach allows for correction of bias, which could be introduced by the different testing strategies used to obtain the LC_50_ or IC_50_ values [44]. Assays were grouped according to the parasite life cycle-stage (epimastigote, trypomastigote or amastigote), duration of incubation with drug (24, 48, 72, 96 or 120 hours) and DTU. Groups with only one assay were excluded from further analysis. For each assay, variables selected (S1 Table) to be run by single means meta-analysis were: parasite stage, DTU, LC_50_ or IC_50_ mean values, standard deviation of LC_50_ or IC_50_ mean values and number of replicates. The “metamean” function for meta-analysis of single means in the *meta* package [45] in R software (version 3.6.1) [46] was used to calculate an overall mean of LC_50_ or IC_50_ values per DTU from each group of assays, using the inverse variance method for pooling, known as the DerSimonian and Laird method [47] and their corresponding ninety-five percent confidence interval. Heterogeneity and publication bias were assessed by constructing funnel plots **(**a scatterplot of treatment effect against a measure of study precision) that plots on x-axis LC_50_ or IC_50_ mean values for each assay against on y-axis a measure of their variability (here, standard error) using the “funnel” function in the *metaphor* package [48]. Subsequently, a meta-regression analysis was performed using the “metareg” function (a wrapper function for “rma.uni” in the *metafor* package [48]) to explore pairwise differences between DTUs within groups defined by parasite form and drug incubation time. Results were considered significant when *p* < 0.05. As recommended by Shild and Voracek [49], we used a rainforest plot, an enhanced variant of the classic forest plot, consisting in a graphical display of estimated results of a number of studies on the same issue (here, the overall average means of LC_50_ or IC_50_ calculated per DTU-parasite form-time point), using the “viz rainforest” function of the *metaviz* package for R [50]. The results of the statistical comparisons between groups defined by parasite form–drug incubation time—DTU were reported on the rainforest plot.

## Results

### Selected publications and assays

**Fig 1:**
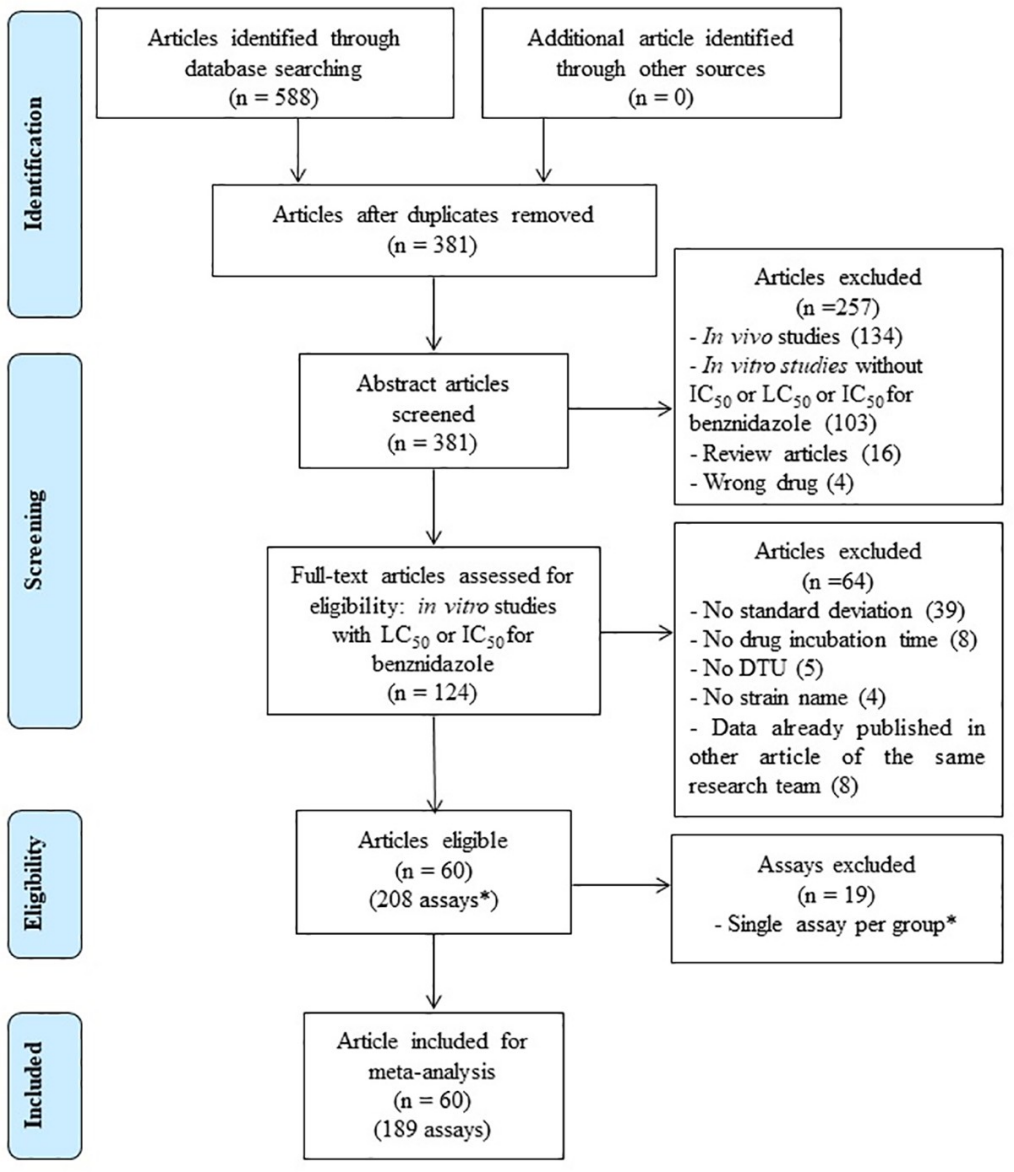
PRISMA flow diagram process for selection of eligible articles and assays. The flow diagram shows the literature search and assays selection process employed for the meta-analysis of benznidazole LC50/IC50 mean values for different T. cruzi DTUs. *If a single assay was available for a given “parasite stage/drug incubation time/DTU” combination, it was deleted from the meta-analysis.

Fig 1 shows the article-selection process. From 588 publications initially identified, 207 were removed due to duplication. From the remaining 381 publications, 257 did not meet the selection criteria: 52.1% measured benznidazole susceptibility only *in vivo*, 40.1% did not report neither the IC_50_ nor the LC_50_ for benznidazole, 6.2% were review articles and 1.6% reported an IC_50_ or an LC_50_ for drugs not including benznidazole. Additionally, from the remaining 124 articles, 64 were excluded: 60.9% did not include the standard deviation, 12.5% did not include the incubation time with the drug, 7.8% did not mention the *T*. *cruzi* DTU, 6.3% did not mention the strain name, and 12.5% correspond to articles where the values of the LC_50_ or IC_50_ and their corresponding standard deviations were previously published in another article by the same research teams. In the end, 60 articles remained, encompassing IC_50_ and LC_50_ values for benznidazole from 208 assays (S1 Table): 97 performed on epimastigotes, 51 on trypomastigotes, and 60 on amastigotes. Data for a total of 59 *T*. *cruzi* strains belonging to 6 different DTUs were analyzed (40 TcI, 6 TcII, 3 TcIII, 1 TcIV, 5 TcV and 4 TcVI strains). Strains originated from seven different countries (Brazil 26, Colombia 15, Chile 1, Mexico 10, Venezuela 3, Argentina 2, Bolivia 1, and Nicaragua 1). For 26.9% of assays the authors used transfected strains or strains cloned in the laboratory. Overall, 11 different methods were used to assess the viability of the parasites; however, manual counting under the *microscope (63*.*6%)*, was the most prevalent. Among the 60 articles, the large majority included only one or 2 assays (63.3%), 23.3% included 3 to 5 assays, and 13.3% a greater number, ranging from 6 to 27.

### Epimastigotes

Table 1 summarizes the information obtained from the 33 articles that included 97 assays (IC_50_ values) for epimastigotes from 49 strains distributed by DTU as follows: TcI 69.4%, TcII 12.2%, TcIII 4.1%, TcV 8.2% and TcVI 6.1%; note that information for TcIV strains was not available. Most of the strains were from Brazil 42.9%, Colombia 28.6%, and Mexico 20.4%, while data for only one strain from Bolivia, Chile, Nicaragua, and Venezuela (2.0% each) could be included in the analysis. In the different studies, parasite viability was primarily determined via microscopic counting (76.3%), while no other method exceeded 8.2%.

**Table 1:** Descriptive information for the 97 available assays of epimastigote susceptibility to benznidazole among the *T*. *cruzi* DTUs.

*T*. *cruzi DTU*	No. articles	No. assays	No. strains	No. countries of origin	No. method	% manual counting^a^
TcI	15	42	34	5	4	85.7
TcII	22	36	6	1	4	75.0
TcIII	2	2	2	1	2	50.0
TcV	6	6	4	2	3	50.0
TcVI	10	11	3	3	4	63.6
Total	33	97	49	7	5	76.3

^a^ Percentage of assays determining parasite viability using a Neubauer cell counting chamber.

### Trypomastigotes

Table 2 summarizes the information obtained from the 31 articles that included 51 assays for trypomastigotes from 14 strains belonging to only three DTUs: TcI 71.4%, TcII 7.1% and TcVI 21.4%. About one-half of the strains were from Brazil (42.8%), while the others were from Mexico, Chile, Colombia and Venezuela. Note that although 24 assays involving TcII were available, they were all performed with the Y-strain. Similar to what we found on epimastigotes, the most common method for measuring parasite viability was microscopic counting (72.5%), either by using a Neubauer counting chamber (56.9%) or by analysis of microscopic fields according to the Brener method (15.7%) [51].

**Table 2:** Descriptive information for the 51 available assays of trypomastigote susceptibility to benznidazole among the *T*. *cruzi* DTUs.

*T*. *cruzi DTU*	No. articles	No. assays	No. strains	No. countries of origin	No. method	% manual counting^a^
TcI	9	18	10	4	4	72.2
TcII	22	24	1	1	3	87.5
TcVI	5	9	3	3	4	33.3
Total	31	51	14	6	7	72.5

^a^ Percentage of assays determining parasite viability using a Neubauer cell counting chamber and the Brener method [51].

### Amastigotes

Table 3 summarizes the information obtained from the 30 publications that included 60 assays for amastigotes from 17 strains mostly distributed into TcI 64.7%, followed by TcVI (2 strains, 11.8%), and only one each (5.9%) for DTUs TcII, TcIII, TcIV and TcV. The strain distribution by country was: Colombia 52.9%, Brazil 23.5%, Venezuela 11.8%, and only one strain from Argentina, Bolivia and Chile (5.9% each). Three main methods for measuring the number of amastigotes per infected cell were used, high-content imaging analysis (40%), microscopic counting after giemsa staining (35.0%), and the colorimetric method using the β-galactosidase-transfected Tulahuen strain (21.7%).

**Table 3:** Descriptive information for the 60 available assays of amastigote susceptibility to benznidazole among the *T*. *cruzi* DTUs.

*T*. *cruzi* DTU	No. articles	No. assays	No. strains	No. countries of origin	No. method	% manual counting^a^
TcI	5	13	11	3	3	69.2
TcII	14	16	1	1	3	68.7
TcIII	1	3	1	1	1	0.0
TcIV	1	3	1	1	1	0.0
TcV	1	3	1	1	1	0.0
TcVI	16	22	2	2	3	9.1
Total	30	60	17	5	5	36.7

^a^ Percentage of assays determining parasite viability using manual counting after giemsa or hematoxylin-eosin staining.

### Meta-analysis of benznidazole LC_50_ or IC_50_ mean values per parasite stage and DTUs

**Fig 2:**
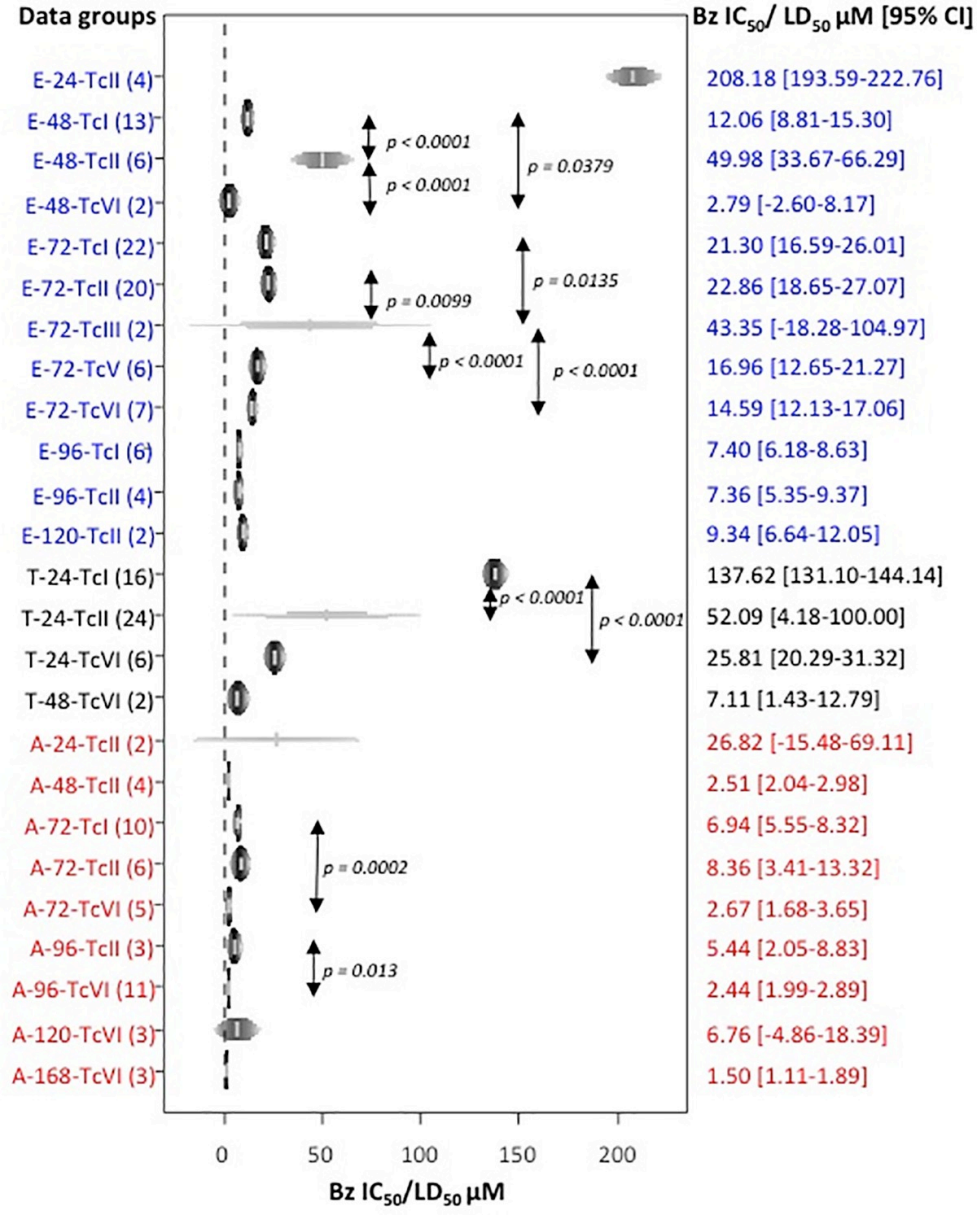
Meta-analysis of benznidazole LC50 and IC50 mean values. Rainforest plot diplaying mean LC50 and IC50 values for benznidazole (Bz) and their 95% confidence intervals (x-axis) estimated by meta-analysis. Combined assays per parasite stage [“E” for epimastigote (blue), “T” for trypomastigote (black) and “A” for amastigote (red)], drug incubation times and DTUs are shown (y-axis); the number of assays per group is displayed in brackets. Mean values and confidence intervals (CI) are shown at the right side of the figure. For comparison of results between DTUs, statistics were applied only between groups belonging to the same parasite stage-incubation time; only significant p-values (< 0.05) are reported.

Fig 2 (rainforest plot) shows LC_50_ or IC_50_ mean values and their 95% confidence intervals for each parasite life-cycle stage, incubation times and DTUs. Since some groups included only one assay, 19 data sets were deleted from the meta-analysis (S2 Table). Ultimately, 189 assays were included.

For epimastigotes, mean IC_50_ values ranged from 2.79 μM (TcVI at 48 hours of incubation) to 208.18 μM (TcII at 24 hours of incubation). There is a general trend for IC_50_ values to decrease as incubation time increases. Nevertheless, this is not true for TcI and TcVI, where the mean IC_50_ values at 72h was superior to the one obtained at 48 hours. At 48 hours of incubation, three DTUs were tested and all pairwise comparisons were significantly different; the susceptibility to benznidazole was TcVI > TcI > TcII showing a very large gap between IC_50_ mean values (2.79 μM vs. 49.98 μM). After 72 hours of incubation with the drug, TcIII appeared significantly less susceptible (43.35 μM [18.28–104.97]) than the other DTUs (TcI, TcII TcV and TcVI), all other pairwise comparisons being not significant. At 48 hours and 72 hours, a larger number of strains were tested for TcI; at 48 hours, 13 TcI strains were included, while for TcII only the Y reference strain was tested and two for TcVI; at 72 hours, 16 TcI strains were tested, while 6 Brazilian strains were included for TcII, 4 for TcV and two for TcIII and TcVI. At 96 hours of incubation with benznidazole, no significant difference between TcI and TcII was found.

Despite the relevance of trypomastigotes for human infection, assays for only three DTUs (TcI, TcII and TcVI) fulfilled the inclusion criteria. Trypomastigote susceptibility was generally tested after a 24 hours incubation with benznidazole. At 48h of incubation, two assays for TcVI performed with Tulahuen strain showed, as expected, a lower mean of LC_50_ value than that obtained for TcVI at 24 hours with three different strains. Overall, at 24 hours of drug incubation, LC_50_ mean values for trypomastigotes ranged from 25.81 μM for TcVI to 137.62 μM for TcI. TcI strains were significantly less susceptible than TcII (*p* < 0.001) and the TcVI (*p* < 0.001) strains. No significant differences were found between TcII and TcVI LC_50_ mean values. It is noteworthy that the analysis yielded a wide confidence interval for TcII strains, although the 24 assays recorded from 22 articles were performed employing the Y-strain exclusively. The reported LC_50_ for Y-strain ranged from 3.07 μM to 282 μM, although parasite viability was measured by microscopic counting using a Neubauer chamber in most (87.5%) cases.

For TcVI amastigotes, assays at 24, 120 and 168 hours incubation with benznidazole were included; the IC_50_ mean value was highest at 24 hours. Most tests were performed after 72 or 96 hours of incubation, and the corresponding IC_50_ mean values range from 2.44 μM (TcVI at 96 hours of incubation) to 8.36 μM (TcII at 72 hours of incubation). TcI strains showed to be significantly less susceptible than TcVI strains at 72 and 96 hours of incubation (*p* < 0.01 and *p* < 0.05 respectively). No other significant pairwise difference was found at 72 or at 96 hours of incubation. Once again, all assays included in the meta-analysis for TcII were performed with the Y-strain. For TcVI only two strains were tested (Tulahuen and CL Brener) while data for 10 TcI strains were available.

Additionally, funnel plots were constructed for each data group (“parasite stage/drug incubation time/DTU”, S1, S2 and S3 Figs). In all cases, data sets did not fit the expected inverted funnel shape and an asymmetrical distribution of values was recorded in general.

## Discussion

### Why a *systematic review and* meta-analysis is relevant to address the question of the impact of genetic variability of *T*. *cruzi* strains over drug sensitivity?

Addressing the possible association between *T*. *cruzi* genetic variability and drug sensitivity requires choosing a drug, an infection model, and a statistical approach. As opposed to nifurtimox and drug candidates currently under study, studies about the susceptibility of *T*. *cruzi* strains to benznidazole on both *in vitro* and *in vivo* models, abound. Including *in vivo* studies in our analysis was not possible because of the high variability of experimental parameters (disease phase, mouse strains, drug administration routes, dosage, treatment duration, and diagnostic tools) impeded proper analysis. Therefore, a meta-analysis using data from published *in vitro* studies seemed more realistic. However, separating the data corresponding to each stage of parasite life cycle, i.e. epimastigotes, trypomastigotes, and amastigotes, was necessary. Additionally, *in vitro* studies provide standard indices for drug sensitivity; namely LC_50_ for the non-replicative trypomastigotes, and IC_50_ for the replicative forms, epimastigotes and amastigotes. Aggregating the values reported for these indices from several independent assays on strains belonging to different DTUs via meta-analysis provides higher statistical power [52]. It is worth mentioning that grouping data was necessary since 63.3% of the 208-recorded assays came from publications where only one or two assays were performed.

### Are there significant statistical differences of susceptibility to benznidazole between DTUs?

Cumulative knowledge regarding the population genetics of *T*. *cruzi* led to a consensual classification into DTUs [14]. This classification constitutes, in our opinion, the most rational basis to explore the impact of the genetic variability of *T*. *cruzi* over parasite intrinsic properties as well as over the parasite´s relationship with its environment. The analysis presented herein unveiled several cases of significant differences in mean LC_50_ or IC_50_ for benznidazole between TcI strains and strains belonging to TcII, TcIII, TcV or TcVI, for all *T*. *cruzi* life cycle stages. Especially striking was the much higher tolerance for benznidazole found for TcI trypomastigotes (drug incubation time of 24h) (LC_50_ = 137.62 μM) in comparison to TcII (LC_50_ = 52.09 μM) and TcVI (LC_50_ = 25.81 μM). In two previous studies, similar trends were observed with epimastigotes of 19/20 strains (TcI) which were found to be less sensitive to benznidazole than 32 and 39 strains (TcII and TcV respectively) [41,53]. Additionally, DTU TcIII, only tested for epimastigote form at 72h of incubation, appeared more tolerant to benznidazole than other DTUs, with significant differences with TcI, TcII, TcV and TcVI DTUs. However, these interesting results must be taken with caution because (i) for the different parasite stages, the numbers of TcII, TcVI and TcIII strains were very low compared to that of TcI, which could influence our findings: e.g. for both trypomastigotes and amastigotes, only one strain was tested for TcII (Y-strain), and two or three for TcVI (CL Brener, Tulahuen and RA), for TcIII only two strains were tested (ii) for epimastigotes significant differences between DTUs were observed after 48h of contact,; however, TcII (not TcI) was less susceptible to benznidazole compared to TcI and TcVI. Our results indicate that benznidazole sensitivity can differ among strains both at the intra- and inter-DTU levels; however, from the data available in the literature, we did not identify a strict correlation between the level of benznidazole tolerance of any given DTU.

### Limitations of the current study

Although interesting trends were identified in the study, asymmetry and dispersion of LC_50_ or IC_50_ mean values outside the pyramid (funnel plot analysis; S1, S2 and S3 Figs) indicate very strong heterogeneity of the data, even within the same parasitic form, DTU, and time of exposure to benznidazole. Even more striking is the very strong heterogeneity of LC_50_ or IC_50_ mean values between assays involving the same strain (S1 Fig (i), epimastigote 72h TcVI see CL strain, S2 Fig (b) trypomastigote 24h TcII see Y strain), which may derive from experimental differences between assays from different articles and laboratories. Indeed, half of the articles in the meta-analysis included a single *T*. *cruzi* life cycle stage (31 out of 60), and most of them (47/60) tested only one or two strains. Additionally, data heterogeneity may arise from the lack of uniformity in the experimental procedures, since the studies have been carried out using different sources of parasite stages (e.g. for trypomastigotes, bloodstream forms from infected mice or cellular forms, for amastigotes from different host cell lines), culture medium, benznidazole sources (pure active compound or commercial pills), benznidazole solublization procedure, as well a variety of methods to determine parasite viability. As a result, available data were extracted from numerous articles and required grouping for further analysis. However, most of the groups of assays included in the meta-analysis were composed by fewer than 10 assays, and constitute a source of dispersion as the estimated mean values are less precise than when larger number of assays are analyzed [54]. All these factors certainly are an important source of the variation reported in LC_50_ and IC_50_ values and their standard deviations, directly affecting the dispersion recorded on the funnel plots [55]. Perhaps because axenic culture is simple and cost-effective, most studies focus on epimastigotes instead of on mammalian-infective forms, which are relevant to human infection. Around 20% of the recorded articles focused on epimastigotes exclusively, and twice as many used assays corresponded to epimastigotes than to trypomastigotes or amastigotes. The published information for *in vitro* benznidazole susceptibility is heavily skewed towards TcI (35.1%) and strains from Brazil. Most of the individual assays recorded in the current study corresponded to TcI, TcII, and TcIV (91.8%). Consequently, the pairwise comparisons of the LC50 or IC50 mean values between DTUs were possible for all three developmental stages of the parasite for only these three DTUs, although with a low sample size in some cases. Data for TcIII were available only for epimastigotes, while no data meeting the inclusion criteria was available for TcIV. In sum, the scarcity of available data prevents an exhaustive exploration of our research question. Additionally, DTU TcII was represented by a single strain (Y- strain) for both mammalian life cycle stages, amastigotes and trypomastigotes. While it is a commonly used reference strain with a single origin, its circulation in countless laboratories over the years could have exposed it to contamination with other strains, as previously demonstrated [56]. It is impossible to ascertain whether such events constitute an underlying cause of the heterogeneity of the LC_50_ and IC_50_ values reported for the Y strain in the literature. This variability highlights the crucial need for Standard Operating Procedures (SOPs) when screening for potential anti-Chagas compounds for each developmental stage.

### Putative sources of variation in benznidazole resistance between *T*. *cruzi* strains

Although specific genes have been implicated in experimentally-induced resistance to benznidazole [57,58,59], natural drug-resistance in *T*. *cruzi* strains is more likely linked to multigene mechanisms [41,59]. For example, transcriptomic analyses of naturally benznidazole-sensitive vs. resistant clones derived from a TcI parental strain revealed differential expression of 133 genes with diverse functions [60]. Additionally, whole genome sequencing of seven Brazilian TcII strains isolated from patients revealed significant intra-DTU genomic variability and aneuploidy, originating from recombination events, mitochondrial introgressions, and chromosomal gain/loss [61]. These underlying genetic differences between closely related strains may be associated to drug-resistance.

## Conclusion and perspectives

Despite the high heterogeneity of our data, our meta-analysis clearly shows that susceptibility to benznidazole *in vitro* differs among *T*. *cruzi* strains. Although intra-strain differences are not constant in every life cycle stage, and cannot be generalized to DTUs, the variation encountered is intriguing and further testing under conditions that allow unbiased comparisons between DTUs is warranted. A lower susceptibility of TcI trypomastigotes to benznidazole (the most noticeable result) could affect cure rates in numerous patients, since TcI is the most widespread *T*. *cruzi* DTU [32]. Despite the uneven representation of data for each DTU in our study, the overall results highlight the need to consider the genetic variability of *T*. *cruzi* during drug optimization, as recently recommended [38]. A higher number and variety of strains must be included during *in vitro* drug screening assays, especially for DTUs TcI, TcII, TcV and TcVI, often associated with human infections.

Lack of uniform methodology also limits our ability to generalize the findings. The advent of automated high-content imaging [62–65], as an alternative for screening of anti-*T*. *cruzi* drugs, allows for large-scale experiments, in particular for the intracellular amastigote model which was recommended to be used as gold standard by the Drugs for Neglected Diseases initiative (DND*i*) [62]. This method, which displays increased sensitivity when compared with colorimetric and fluorometric assays [38,62], will allow to screen large compound libraries for activity against standard *T*. *cruzi* strains and field isolates representative of the different DTUs. Efforts should be made to deploy this technology to endemic countries.

## Supporting information

S1 Fig Funnel plots for assays measuring epimastigote susceptibility to benznidazole. Scatterplots show the mean IC_50_ values for each assay on the x-axis and their standard errors in the y-axis. In the absence of both heterogeneity and publication bias, 95% of assays would lie in the region below the straight lines. Each scatterplot included assays performed with strains belonging to the same DTU at a given time of incubation with benznidazole: (a) 24h, (b—d) 48h, (e–i) 72h, (k) 96h, and (l) 120h.
(DOCX)

S2 Fig Funnel plots for assays measuring trypomastigote susceptibility to benznidazole. Scatterplots show the mean LC_50_ values for each assay on the x-axis and their standard errors in the y-axis. In the absence of both heterogeneity and publication bias, 95% of assays would lie in the region below the straight lines. Each scatterplot included assays performed with strains belonging to the same DTU at a given time of incubation with benznidazole: (a—c) 24h, and (d) 48h.
(DOCX)

S3 Fig Funnel plots for assays measuring amastigote susceptibility to benznidazole. Scatterplots show the mean IC_50_ values for each assay on the x-axis and their standard errors in the y-axis. In the absence of both heterogeneity and publication bias, 95% of assays would lie in the region below the straight lines. Each scatterplot included assays performed with strains belonging to the same DTU at a given time of incubation with benznidazole: (a) 24h, (b) 48h, (c—e) 72h, (f) and (g) 96h, (h) 120h, and (i) 168h.
(DOCX)

S1 Table Details of each benznidazole susceptibility assay for *T*. *cruzi* strains recorded from 60 selected articles for the meta-analysis. (XLSX)

S2 Table Number of recorded assays per parasite stage, DTU and drug incubation time. (XLSX)
