# Supplementary figures and images for: In vitro susceptibility of Trypanosoma cruzi discrete typing units (DTUs) to benznidazole: A systematic review and meta-analysis
Source: PLoS Negl Trop Dis. 2021 Mar 22;15(3):e0009269. doi: 10.1371/journal.pntd.0009269 (PMC8016252; doi:10.1371/journal.pntd.0009269)

1.
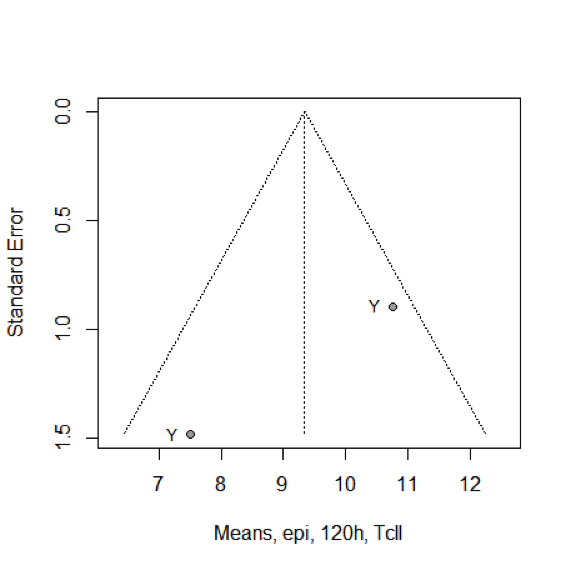

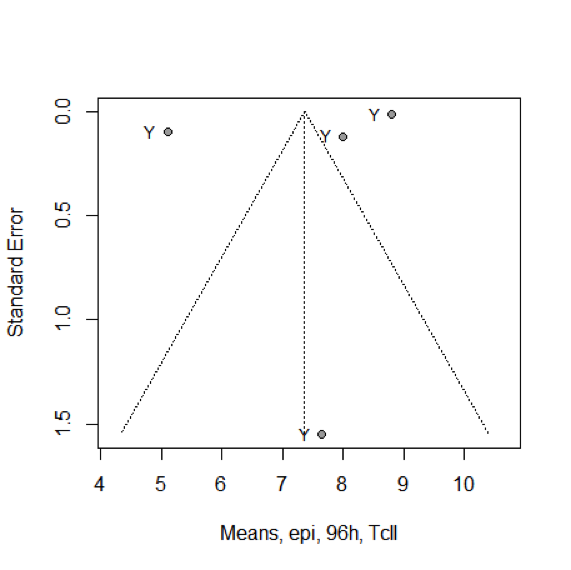

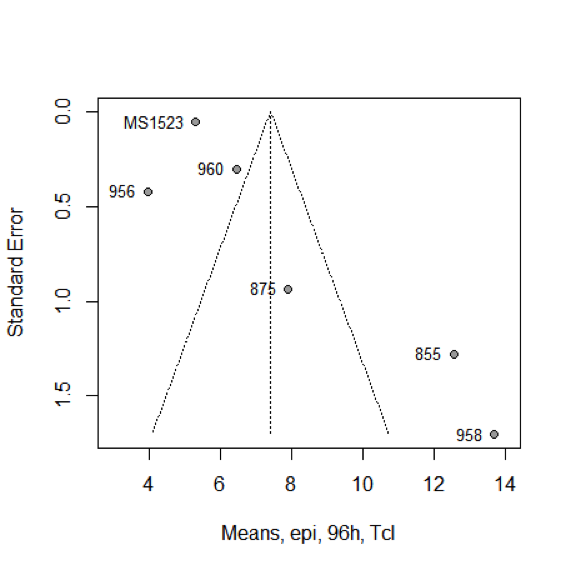
b) c)


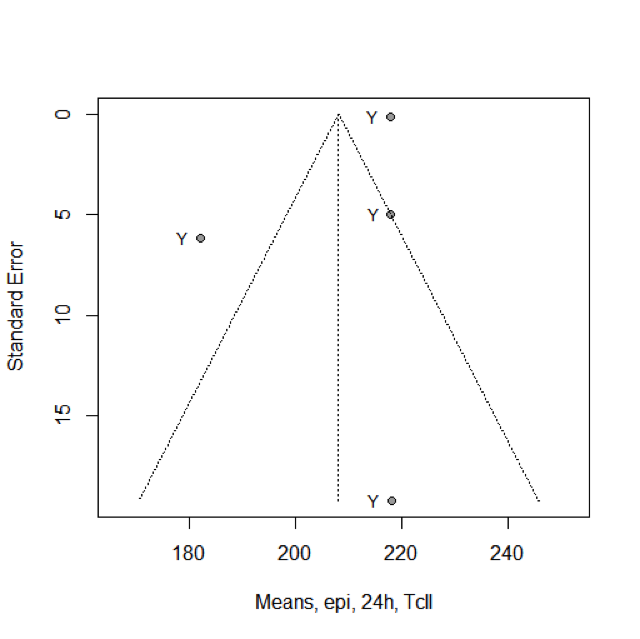

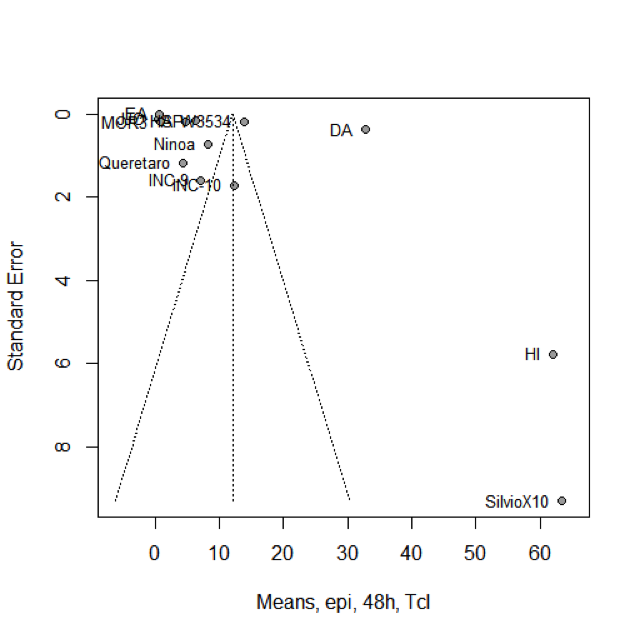

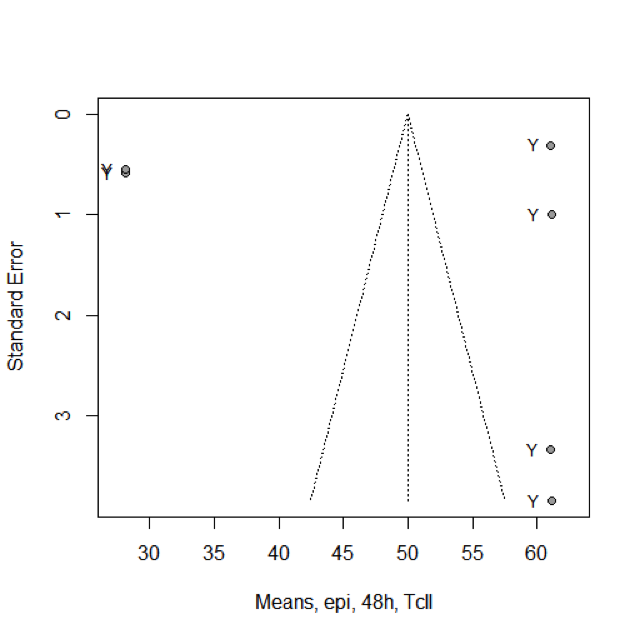

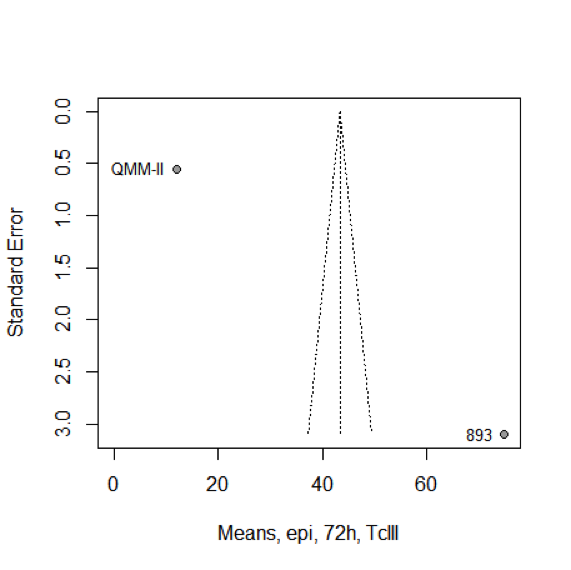

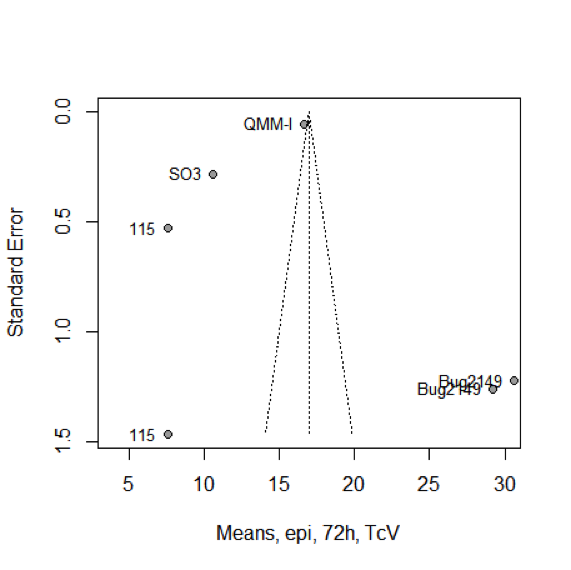

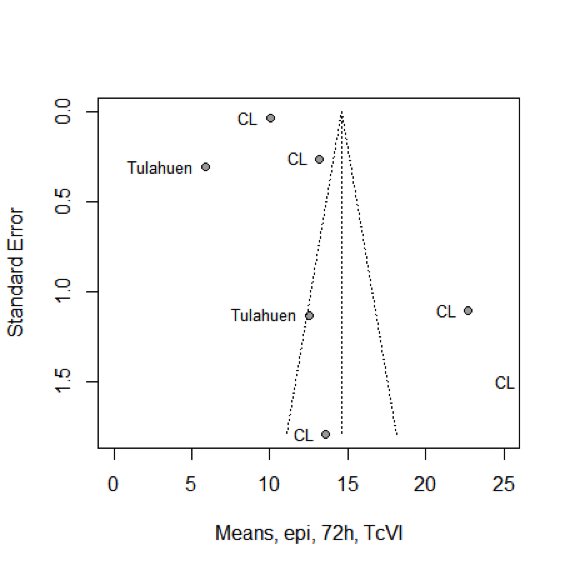

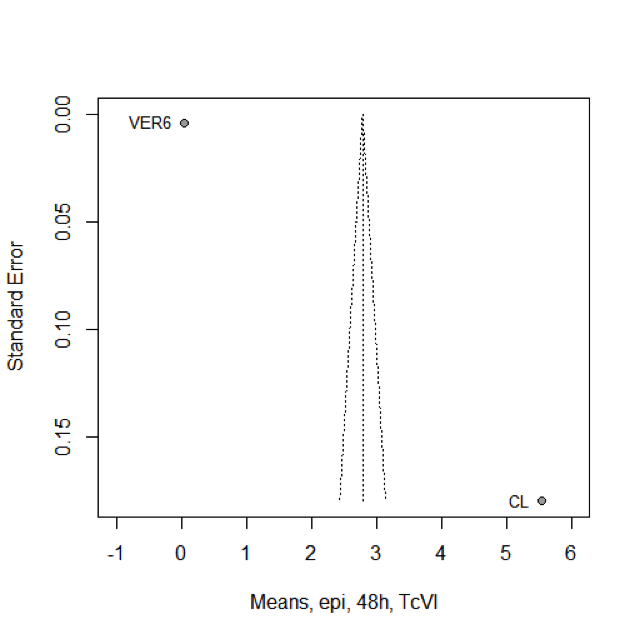

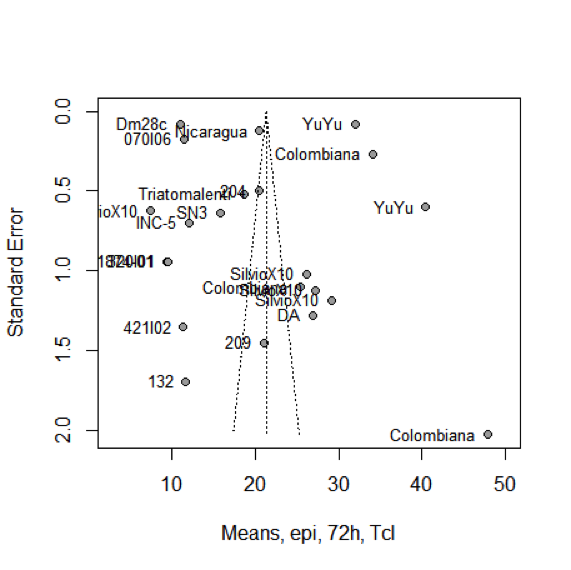

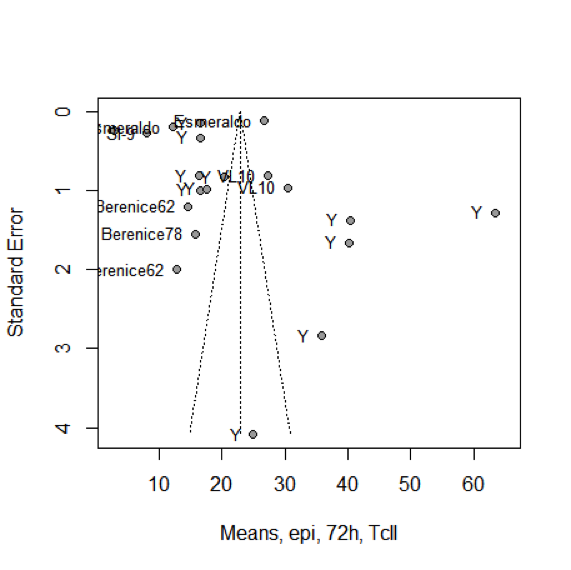


d) e) f)

g) h) i)

j) k) l)

Supplement: S1 Fig — Scatterplots show the mean IC50 values for each assay on the x-axis and their standard errors in the y-axis. In the absence of both heterogeneity and publication bias, 95% of assays would lie in the region below the straight lines. Each scatterplot included assays performed with strains belonging to the same DTU at a given time of incubation with benznidazole: (a) 24h, (b—d) 48h, (e–i) 72h, (k) 96h, and (l) 120h. (DOCX) [file pntd.0009269.s001.docx]

1. b)


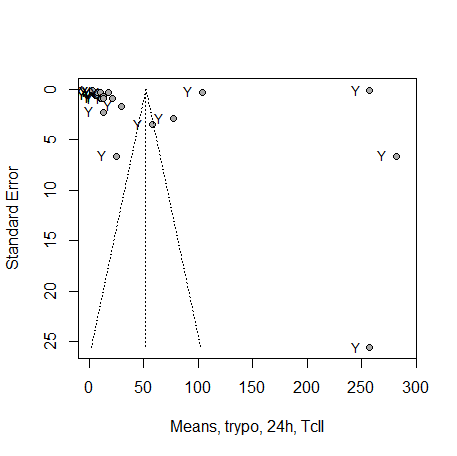

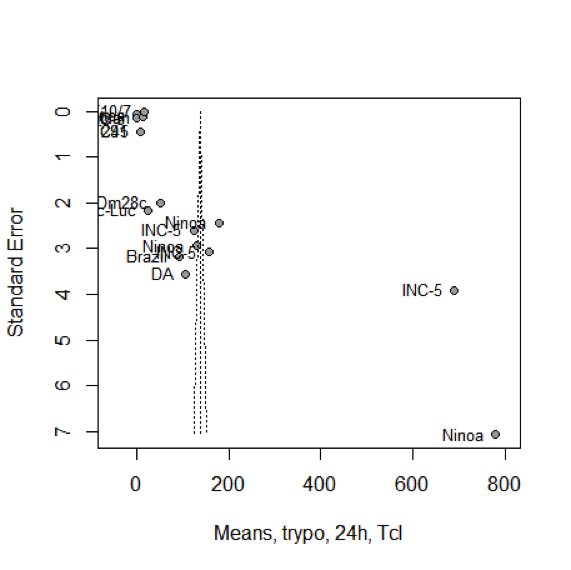

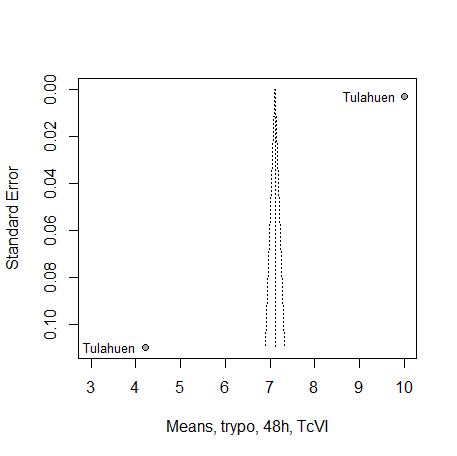

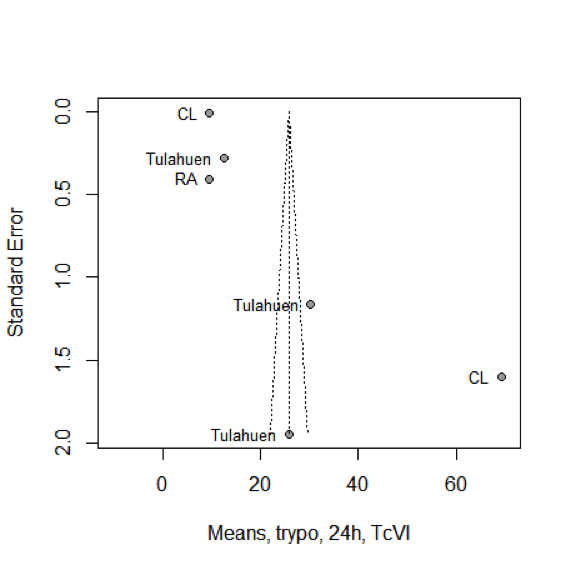


c) d)

Supplement: S2 Fig — Scatterplots show the mean LC50 values for each assay on the x-axis and their standard errors in the y-axis. In the absence of both heterogeneity and publication bias, 95% of assays would lie in the region below the straight lines. Each scatterplot included assays performed with strains belonging to the same DTU at a given time of incubation with benznidazole: (a—c) 24h, and (d) 48h. (DOCX) [file pntd.0009269.s002.docx]

a) b) c)


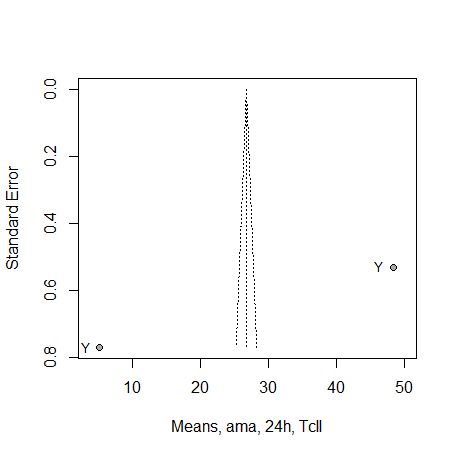

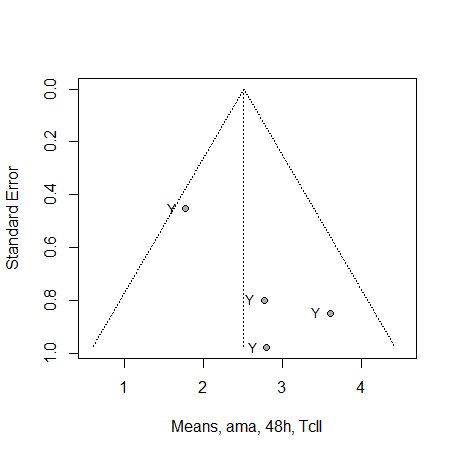

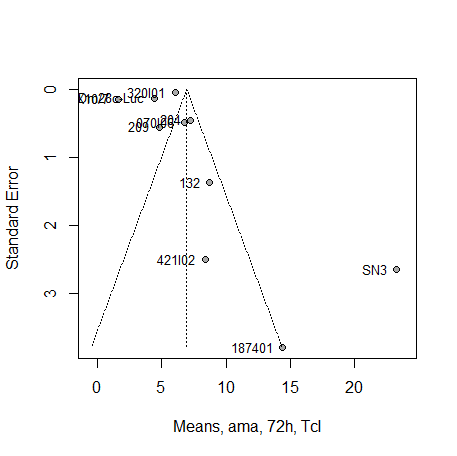

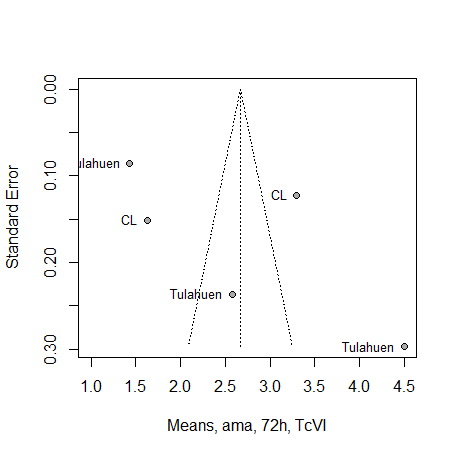

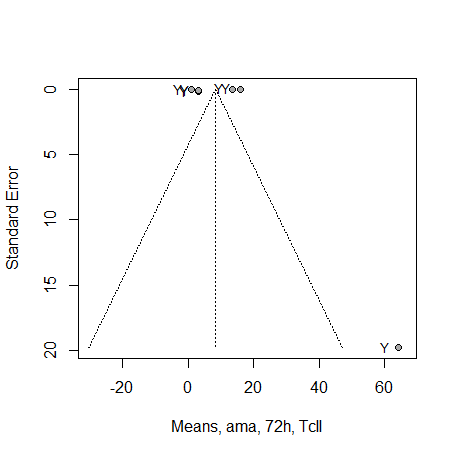

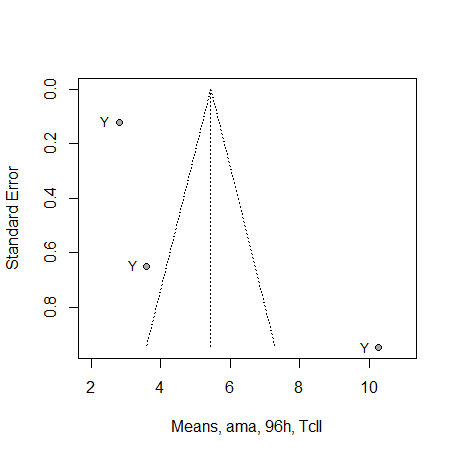


d) e) f)


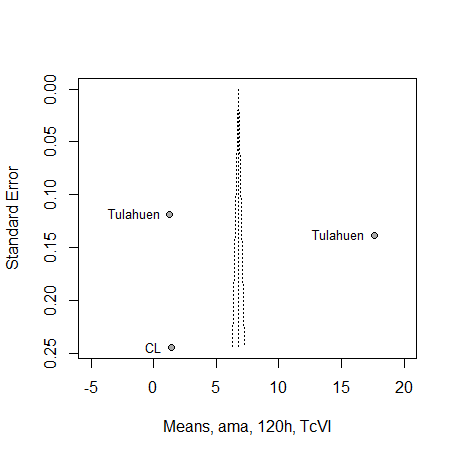

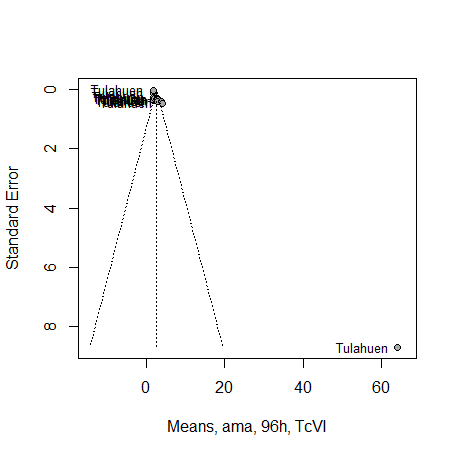

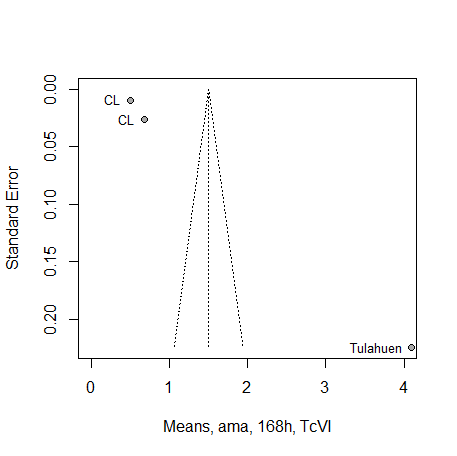


g) h) i)

Supplement: S3 Fig — Scatterplots show the mean IC50 values for each assay on the x-axis and their standard errors in the y-axis. In the absence of both heterogeneity and publication bias, 95% of assays would lie in the region below the straight lines. Each scatterplot included assays performed with strains belonging to the same DTU at a given time of incubation with benznidazole: (a) 24h, (b) 48h, (c—e) 72h, (f) and (g) 96h, (h) 120h, and (i) 168h. (DOCX) [file pntd.0009269.s003.docx]
